# Non-invasive alternative for phosphodiesterase inhibitor-refractory erectile dysfunction: Real-life experience with low-intensity extracorporeal shockwave therapy

**DOI:** 10.1097/MD.0000000000035939

**Published:** 2023-11-10

**Authors:** Necmi Bayraktar

**Affiliations:** a Burhan Nalbantoğlu State Hospital, TRNC, Nicosia, Turkey.

**Keywords:** comorbidities, erectile dysfunction, low-intensity extracorporeal shockwave therapy, phosphodiesterase inhibitors, treatment efficacy

## Abstract

This retrospective study assessed the efficacy of low-intensity extracorporeal shockwave therapy (Li-ESWT) in the treatment of erectile dysfunction (ED) in patients unresponsive to phosphodiesterase inhibitors (PDE5is). Between May 2020 and December 2022, we retrospectively analyzed the records of 126 ED patients who underwent Li-ESWT post unsuccessful PDE5is trials, defined as inadequate response following at least 6 consistent trials with correct dosage (preference given to 20 mg tadalafil). Patients with neurogenic disorders were excluded. Patients’ ED severity was determined using the IIEF-5 score and further categorized into 2 groups. The Li-ESWT treatment protocol consisted of 12 weeks. Data was analyzed using descriptive statistics and paired t-tests. In the cohort of 126 patients, the mean age was 50.5 ± 12.4 years, with a BMI of 29.18 ± 3.49. Notably, 74.6% had ED for more than 12 months. Before Li-ESWT, 55.6% used sildenafil and 44.4% used tadalafil. Post 3 months of Li-ESWT, the average IIEF score rose significantly from 10.19 ± 7.71 to 14.29 ± 0.92 (*P* < .01). Particularly, Group 2 exhibited a significant improvement in their mean IIEF score from 13.78 ± 1.38 pretreatment to 21 ± 2.31 post-treatment. However, Group 1 (with higher diabetes prevalence) showed a marginal rise from 5.8 ± 1.47 to 6.1 ± 3.2 (*P* = .14). Similarly, the overall EHS score progressed significantly from 1.34 ± 0.8 to 2.3 ± 1.17 post-treatment. Post-treatment, while Group 1 showed no changes in successful vaginal penetration, Group 2 reported a dramatic increase in successes, from 16 before treatment to 68 after. This study demonstrated the efficacy of Li-ESWT for PDE5is-refractory ED, particularly in patients with moderate to mild ED. However, patients with severe ED and comorbidities did not show significant improvement. Further research with larger sample sizes, control groups, longer follow-up periods, and standardized protocols is required to confirm the effectiveness and limitations of Li-ESWT in ED treatment.

## 1. Introduction

Erectile dysfunction (ED) is a common disease in men. Similar prevalence rates have been observed in different countries regardless of age. Although its physiopathology is multifactorial, the most common cause is a vascular disorder. Associated conditions, such as diabetes mellitus, atherosclerosis, concomitant coronary artery diseases, and drug use on a psychological and hormonal basis, play a role in the emergence of the disease.^[1]^ There are many treatment options for organic ED, such as lifestyle alterations, oral pharmacotherapy, intra-cavernosal injections, and penile implants. Low-intensity extracorporeal shockwave therapy (Li-ESWT) has been the most popular noninvasive ED treatment for vasculogenic ED over the past 10 years; however, few studies have been conducted.

Most patients prefer noninvasive methods for ED treatment. Although changing the lifestyle (healthy diet, smoking cessation, regular physical activity, and avoiding obesity) can prevent progression or improve regression of ED, many patients have self-confidence problems and use oral medications.^[2]^ Thus, many patients start oral medicines before or after consulting a physician. Since many factors play a role in the pathophysiology of ED, it is challenging to plan treatment for a single pathology.

For the first time in 2010, Vardi et al^[3]^ reported the effectiveness of ESWT treatment of ED. Li-ESWT therapy provides a noninvasive, safe, and effective ED treatment despite device and settings variations.^[4]^ Many studies and reviews have reported that Li-ESWT will become the first-line therapy in ED treatment.^[5]^ The therapeutic effect of Li-ESWT is to increase blood flow by stimulating angiogenesis and creating new blood vessels.^[6]^ Mechanism demonstrated by increasing vascular endothelial growth factor (VEGF) secretion and improving vascular expression in the corpus cavernosum.^[7,8]^

In cases of ED refractory to PDE5is (phosphodiesterase type 5 inhibitors), switching to surgical or invasive injection therapy is considered. However, the pain associated with injection therapy, the surgical process in the penile implant procedure, and concerns about losing naturalness reduce the willingness for these treatments. Based on the therapeutic efficacy mechanism of Li ESWT in treating ED, as well as published studies spanning a decade, we believe that this treatment could be beneficial for our patients in Northern Cyprus. The recommended treatment protocols were arranged according to the recommendations of the manufacturer and end users and were applied after 2020.

The purpose of our study was to share our real-life experience of treating patients who did not respond to phosphodiesterase inhibitors using Li-ESWT, a noninvasive alternative treatment option.

## 2. Material and methods

### 2.1. Study design

This study retrospectively analyzed the records of 126 patients with ED who underwent Li-ESWT between May 2020 and December 2022. This study primarily sought to determine the efficacy of Li-ESWT therapy in managing ED among patients who failed to benefit from phosphodiesterase inhibitors.

### 2.2. Eligibility criteria

The study retrospectively included patients who had administered any PDE5is before undergoing Li-ESWT therapy and either did not experience any significant benefits or only obtained partial advantages. Patients who had not received any PDE5is inhibitor prior to Li-ESWT therapy, and those diagnosed with neurogenic disorders were excluded.

Through retrospective analysis, this study included 126 patients, each of whom underwent Li-ESWT after a prior unsuccessful treatment with PDE5is that failed to yield the expected response. Prior to the implementation of Li-ESWT, rigorous checks were instituted to ensure that all patients had been correctly utilizing PDE5is, with a preference for 20 mg tadalafil for the prescribed duration. The criteria for PDE5is unresponsiveness were established as a lack of adequate response following at least 6 trials of PDE5is, each adhering to the appropriate dosages and durations.^[9,10]^

### 2.3. Data analysis

Descriptive statistics were used to analyze the characteristic features. Categorical data are expressed as numbers (n) and percentages, whereas quantitative data are presented as mean ± standard deviation (SD). The patient sample was subdivided into 2 groups based on the IEFF-5 score (severe or moderate-mild). A paired *t* test was used to compare the groups of dependent variables. The statistical package program SPSS Windows version 24.0 was used for data analysis.

### 2.4. Ethical considerations

Patients were informed about the Li-ESWT protocol and consent forms were obtained prior to the initiation of treatment. This study adhered to the guidelines outlined in the Declaration of Helsinki and was approved by the Ethics Committee of TRNC Burhan Nalbantoğlu State Hospital Ethics Committee (Issue: Project Code: 08/23).

### 2.5. Treatment and follow-up protocol

Patients reporting ED were thoroughly examined and their detailed medical histories were recorded. Laboratory tests were performed to evaluate the severity of ED. A patient’s inability to achieve erection sufficient for vaginal penetration despite PDE5is treatment was recorded as unresponsive to treatment. Before Li-ESWT, the degree of ED, IEFF-5, Erectile Hardness Score (EHS), morning erections, and success in achieving vaginal penetration were recorded. The Li-ESWT treatment protocol was executed over 6 weeks, with 3 sessions per week for the first 2 weeks without anesthesia, a break of 2 weeks, followed by 6 more sessions in the subsequent 2 weeks. Each session involved 1500 shock waves with a frequency of 5 per second and energy of 0.10 mJ/mm^2^. The Li-ESWT application areas consist of 3 foci (proximal, mid-shaft, and distal parts) on both dorsal corpus cavernosa. All patients continued tadalafil 5 mg daily for 6 weeks during the Li-ESWT Protocol, and for an additional 6 weeks. Patients were verbally encouraged to attempt regular sexual intercourse during the 6-week treatment protocol. The patient’s response to the Li-ESWT protocol was evaluated after 12 weeks through reassessment of IEFF-5 and EHS and successful vaginal penetration.

## 3. Results

In the study sample group, none of the patients opted to discontinue treatment because of pain or other complications. The mean age of the participants included in the study was calculated to be 50.5 ± 12.4 years, with a mean Body Mass Index (BMI) of 29.18 ± 3.49. A significant proportion (74.6%) of patients had been experiencing ED for over 12 months. Prior to the study, all the patients had a history of sildenafil (55.6%) and tadalafil (44.4%). However, 72.2% used PDE5is without consulting a physician. The patient demographics and related characteristics are presented in Table 1.

**Table 1:** Patient demographics and characteristics.

	Patients (Total = 126)	Group 1 (57)	Group 2 (69)
Age (yr), mean ± SD	50.5 ± 12.4	55.38 ± 9.7	53.64 ± 13.88
BMI (kg/m^2^), mean ± SD	29.18 ± 3.49	29.72 ± 3.57	28.26 ± 3.14
ED duration			
More than 6 months, N (%)	32 (25.4)	5 (8.8)	27 (39.1)
More than 12 months, N (%)	94 (74.6)	52 (91.2)	42 (60.9)
Morning erection			
No, N (%)	69 (54.8)	43 (75.4)	26 (37.7)
Yes, N (%)	4 (3.2)	Nil	4 (5.8)
Sometimes, N (%)	53 (42.1)	14 (24.6)	39 (56.5)
Total testosterone (ng/dL), mean ± SD	6.3 ± 3.6	7.65 ± 4.12	5.3 ± 2.5
HbA1c	5.8 ± 1.32	6.48 ± 1.48	5.52 ± 0.91
Total Cholesterol	198.1 ± 40.3	192.6 ± 39.2	202.7 ± 42.5
Triglyceride	164.9 ± 70.5	157 ± 59	171.8 ± 78.6
Smoking history			
Non-smokers, N (%)	70 (55.6)	38 (66.7)	32 (46.4)
Smokers, N (%)	45 (35.7)	14 (24.6)	31 (44.9)
Smokers Who Quit, N (%)	11 (8.7)	5 (8.8)	6 (8.7)
PDE5is treatment (Before Li-ESWT)			
Sildenafil, N (%)	70 (55.6)	30 (52.6)	40 (58)
Tadalafil, N (%)	56 (44.4)	27 (47.4)	29 (42)
Physician recommendation for PDE5is			
No	91 (72.2)	41 (71.9)	50 (72.5)
Yes	35 (28.8)	16 (28.1)	19 (27.5)

BMI = body mass index, ED = erectile dysfunction, HbA1c = Hemoglobin A1c, PDE5is = phosphodiesterase 5 inhibitors, SD = standard deviation.

In our cohort, we observed a 24.6% incidence rate of diabetes, whereas coronary artery disease was reported in 27.4% of the sample. Notably, the distribution of diabetes was highly skewed across the groups. Among those in Group 1, diabetes was prevalent in 25 out of 57 individuals, in stark contrast to Group 2, where only 6 of the 69 participants were diabetic. Table 2 provides a comprehensive breakdown of the prevalence of systemic diseases among the patient groups presented as absolute numbers and percentages.

**Table 2:** Summary of systemic disease history of the patients.

		*N*	%
	No systemic diseases	10	10.5
	Diabetes mellitus	25	26.3
	Hyperlipidemia	9	9.5
	Hypertension	18	18.9
	Coronary artery disease	26	27.4
	Others	7	7.4
Total		95	100.0

Three months after treatment, significant enhancements were observed in the International Index of Erectile Function-5 (IIEF-5) and Erection Hardness Scale (EHS) scores following administration of low-intensity extracorporeal shockwave therapy (Li-ESWT), as shown in Table 3. Collectively, the subjects presented a substantial increase in the average IIEF score, escalating from a pretreatment 10.19 ± 7.71 to 14.29 ± 0.92 post-therapy (*P* < .01). Group 2 mirrored this upward trend, with their mean IIEF score reflecting a considerable boost from 13.78 ± 1.38 before treatment to 21 ± 2.31 after the therapy (*P* < .01). However, Group 1, while demonstrating a slight increase from 5.8 ± 1.47 to 6.1 ± 3.2 post-therapy, did not reach statistical significance (*P* = .14). Similar observations were made for the EHS scores. The overall study group exhibited a significant improvement, with the mean EHS score transitioning from 1.34 ± 0.8 pre-therapy to 2.3 ± 1.17 post-therapy (*P* < .01). Parallel to this, Group 2 showcased a notable enhancement, with the scores rising from 1.71 ± 0.6 to a commendable 3.2 ± 0.7 post-therapy (*P* < .01). Conversely, Group 1, despite a modest elevation from 0.91 ± 0.8 1.2 ± 0.68, was not statistically significant (*P* = .14).

**Table 3:** Pre- and post-Li-ESWT comparative analysis.

	Total		Group 1 N (%) =26(35.6%)		Group 2 N (%) =31(42.5%)	
	mean ± SD	*P* value	mean ± SD	*P* value	mean ± SD	*P* value
Pre Li-ESWT IEFF	10.19 ± 7.71	<0.01*	5.8 ± 1.47	0.14†	13.78 ± 1.38	<0.01‡
Post Li-ESWT IEFF	14.29 ± 0.92		6.1 ± 3.2		21 ± 2.31	
Pre Li-ESWT EHS	1.34 ± 0.8	<0.01§	0.91 ± 0.8	0.14‖	1.71 ± 0.6	<0.01¶
Post Li-ESWT EHS	2.3 ± 1.17		1.2 ± 0.68		3.2 ± 0.7	

EHS = Erection Hardness Scale, IIEF = International Index of Erectile Function-5, Li-ESWT = low-intensity extracorporeal shockwave therapy, SD = standard deviation, VP = vaginal penetration.

* Paired Samples *t* test, Effect size Cohen’d 1 (0.79–1.219 95% CI).

† Paired Samples *t* test, Effect size Cohen’d 0.19 (–0.65–0.459 95% CI).

‡ Paired Samples *t* test, Effect size Cohen’d 3.8 (3.131–4.495 95% CI).

§ Paired Samples *t* test, Effect size Cohen’d 0.9 (0.765–1.190 95% CI).

‖ Paired Samples *t* test, Effect size Cohen’d 0.51 (0.238–0.791 95% CI).

¶ Paired Samples *t* test, Effect size Cohen’d 2.2 (1.802–2.691 95% CI).

Upon analysis conducted 3 months after the cessation of treatment, a persistent trend of zero occurrences of vaginal penetration was observed within Group 1, pointing to a lack of significant change in this critical outcome measure. In contrast, Group 2 exhibited a striking shift in the same metric. Before the intervention, the group consisted of 16 patients failed to achieve vaginal penetration, whereas 53 patients reported successful vaginal penetration. Post-treatment, the results were significantly skewed towards success, with 68 patients reporting successful vaginal penetration, a figure counterbalanced by only 1 patient unable to achieve the same. These results highlight the dichotomous outcomes of the application of low-intensity shockwave therapy (Li-ESWT), underscoring its potential therapeutic efficacy in some patients while simultaneously delineating its limitations in others.

## 4. Discussion

Low-intensity extracorporeal shock wave therapy (LI-ESWT) elicits neovascularization of the erectile tissue with low-intensity acoustic energy to the penile corpora. Theoretically, it is expected to improve and/or restore the erectile function.^[4]^ Comorbidities and associated conditions in ED affect treatment and study results. Therefore, creating homogeneous patient groups for clinical studies is difficult. Unfortunately, our study was small, was based on a retrospective review, and lacked sufficient homogeneity for strong evidence.

Erectile dysfunction presents with a 30% increased prevalence in overweight patients.^[11]^ When we evaluate the BMI values of our 2 patient groups, it becomes apparent that they consist primarily of individuals exceeding the normal weight range. The implications of lifestyle and dietary habits are indeed multifold, influencing not only the risk and management of systemic diseases but also contributing significantly to the development and progression of ED. Therefore, it is crucial to consider these factors in our discussion and treatment strategies for managing ED.^[12]^

Diabetes is a well-established leading cause of emergency dysfunction.^[13]^ Diabetes Mellitus (DM), a significant risk factor in the etiology of ED, was diagnosed in a substantial 26.3% of our cohort. Our study echoes findings from the broader literature, including the work of Vardi et al, which suggests reduced efficacy of Li-ESWT in patients with diabetes, particularly in those with severe ED.^[14]^ This trend was evident in our study, where we observed a significantly less beneficial response to Li-ESWT in Group 1, a cluster characterized by severe ED and elevated HbA1c levels. In contrast, groups showing a more favorable response to therapy, such as Group 2, typically displayed HbA1c levels within the normal range. Notably, despite controlled diabetes, Group 1 did not demonstrate substantial improvement in vaginal penetration post-Li-ESWT, underscoring the need for more tailored treatment strategies for this patient subset. This conclusion contributes to the current body of literature by highlighting the potential influence of diabetes on Li-ESWT outcomes, thus offering a valuable perspective for future clinical and research endeavors.

Coronary artery disease is another prominent etiological factor for ED. Recent research has shed light on the capacity of PDE5is to reduce mortality in cardiovascular disease while exerting pleiotropic effects in diabetic patients.^[15]^ Animal studies further illuminated the beneficial effects of Li-ESWT in chronic ischemic diseases.^[6,16]^ For patients with coronary artery disease presenting with PDE5i-refractory ED, Li-ESWT not only offers a potential treatment pathway for ED but may also facilitate the reestablishment of regular PDE5i usage. The efficacy of Li-ESWT in cases of PDE5i-refractory non-severe ED has been underscored in numerous studies.^[14]^ In our analysis, coronary artery disease did not emerge as a significant adverse risk factor in response to Li-ESWT, in contrast to the observed outcomes in patients with diabetes. Our results suggest that Li-ESWT can potentially restore erectile function and enable beneficial responses to PDE5is in this patient group. However, these findings require further validation.

The efficiency of Li-ESWT in moderate and mild ED has been demonstrated in several clinical studies.^[3,17,18]^ In addition, the effectiveness of Li-ESWT for ED was determined.^[16,19]^ Li-ESWT device differences and the effects of the differences between the application protocols on the results are discussed separately. One of the limitations of our study was that we did not conduct a comparative study on this subject.

It is well known that Li-ESWT is effective in moderate and mild ED unresponsive to PDE5is.^[14,20]^ In our study, the PDE5is refractory patients in this group showed an improvement in response to PDE5is and Erectile dysfunction after Li-ESWT. Differences in the IEFF-5 and EHS pre- and post-treatment scores demonstrated this response. Although there was minimal improvement in the IEFF-5 scores and EHS in the group with chronic systemic diseases and severe ED, successful vaginal penetration could not be achieved. Our outcomes are supported by the similar results of similar studies conducted previously.^[14,21,22]^

### 4.1. Study limitations

This study had several important limitations. The retrospective design creates uncertainties and can potentially lead to incomplete or misleading patient records. In addition, the study population was relatively small, which could limit the statistical power and generalizability of the findings. The lack of a control group also means that the results could be influenced by factors other than Li-ESWT treatment, such as the natural progression of ED or concurrent therapies that the patients were undergoing. Furthermore, the follow-up period was relatively short, which may not adequately capture the long-term outcomes of the Li-ESWT. Finally, self-reported patient measures such as the IIEF-5 score may have been subject to recall bias and subjective interpretation, potentially affecting the reliability of the results.

Despite these limitations, our study provides valuable insights into the real-life experience and outcomes of patients treated with Li-ESWT for PDE5is-refractory ED. Future research could benefit from a larger sample size, control group, and longer follow-up period to provide more robust evidence of the effectiveness and potential limitations of Li-ESWT in ED.

## 5. Conclusion

Our findings suggest that low-intensity extracorporeal shockwave therapy (Li-ESWT) may serve as a promising therapeutic option for patients with moderate-to-mild ED unresponsive to phosphodiesterase type 5 inhibitors (PDE5is). However, our results indicate that patients with severe ED, particularly those with uncontrolled diabetes, may benefit less from this therapy. These findings underscore the importance of patient selection and comorbidity control in enhancing the efficacy of Li-ESWT, and contribute to the expanding body of knowledge guiding ED management. It is also important to highlight the need for further research, particularly large-scale randomized controlled trials, to confirm these findings and establish more definitive guidelines for the application of Li-ESWT in the management of ED, particularly in patients who are unresponsive to conventional PDE5is therapy.

## Author contributions

**Conceptualization:** Necmi Bayraktar.

**Data curation:** Necmi Bayraktar.

**Formal analysis:** Necmi Bayraktar.

**Funding acquisition:** Necmi Bayraktar.

**Investigation:** Necmi Bayraktar.

**Methodology:** Necmi Bayraktar.

**Resources:** Necmi Bayraktar.

**Supervision:** Necmi Bayraktar.

**Writing – original draft:** Necmi Bayraktar.

**Writing – review & editing:** Necmi Bayraktar.
